# Gestational Low Protein Diet Increases the Susceptibility to *N*‐Bis(2‐Hydroxypropyl)nitrosamine‐Induced Lung Tumors in Female Wistar Rats

**DOI:** 10.1002/em.70086

**Published:** 2026-10-24

**Authors:** Vanessa de Cássia Martins Pires Ferreira, Victor Hugo Carvalho, Patrícia Aline Boer, Luís Fernando Barbisan

**Affiliations:** ^1^ Department of Cellular and Molecular Biology Institute of Biosciences, UNESP—São Paulo State University Botucatu SP Brazil; ^2^ Department of Internal Medicine, Nucleus of Medicine and Experimental Surgery, FCM UNICAMP—State University of Campinas Campinas SP Brazil

**Keywords:** female Wistar rats, fetal programming, lung and kidney carcinogenesis, maternal low protein intake, pulmonary epithelial DNA damage

## Abstract

This study evaluated the susceptibility to lung and kidney carcinogenesis induced by *N*‐bis(2‐hydroxypropyl)nitrosamine (DHPN) in female rats whose dams received a low‐protein diet (LPD) during gestation. Pregnant Wistar rats were fed normal protein diet (NPD, 17% protein) or LPD (6% protein). After parturition, all dams were fed NPD until weaning. Then, female offspring were distributed into four groups (NPD, LPD, NPD + DHPN, and LPD + DHPN) and fed NPD. At 6 weeks old, two groups received 0.2% DHPN in drinking water for 4 weeks, and some females were euthanized 24 h after carcinogen stopping, while others were euthanized at Week 26. First, the lung was removed, fixed, histologically processed, and tissue sections were immunohistochemically stained for γ‐H2AX. Second, the lung and kidneys were removed, fixed, histologically processed, and hematoxylin–eosin stained tissue sections were obtained for histopathological analysis. Female rats whose dams received LPD developed a higher mean number of pulmonary epithelial cells positive for γ‐H2AX (*p* < 0.005) and bronchioloalveolar hyperplasia and adenomas (*p* < 0.001 and *p* < 0.001) in the lung compared with their counterparts. LPD did not alter the development of atypical tubule hyperplasia in the kidney. These findings indicated that LPD increased DHPN‐induced lung carcinogenesis in female rats.

## 1 Introduction

The developmental origins of health and disease (DOHaD) concept is supported by a large body of epidemiological and experimental studies demonstrating that maternal malnutrition can result in developmental imprinting and fetal epigenetic reprogramming, thereby increasing susceptibility to several chronic diseases later in life (Barker 2007; Ramakrishnan et al. 2014; Herring et al. 2018; Fioretto et al. 2025). Protein–energy malnutrition during pregnancy remains a major contributor to intrauterine growth restriction (IUGR) in developing countries (Ramakrishnan et al. 2014). In IUGR rodent studies, gestational low‐protein intake is associated with functional and morphological disorders in the kidney (Rizzi et al. 2017; Sene et al. 2018), prostate (Rinaldi et al. 2013; Pinho et al. 2014), heart (Assalin et al. 2019; Folguieri et al. 2022), lung (Zana‐Taieb et al. 2015; Gil et al. 2021), and other organs in the offspring. In addition, some studies have suggested that gestational and lactational low‐protein intake can increase susceptibility to spontaneous or chemically induced tumors in the prostate and mammary gland of offspring, respectively (Santos et al. 2019; Zapaterini et al. 2022).

*N*‐bis(2‐hydroxypropyl)nitrosamine (DHPN), a potent mutagen and broad‐spectrum carcinogen, has been shown to induce tumors in the nasal cavity, lung, thyroid, liver, kidney, and urinary bladder of both sexes across various rat strains (Konishi et al. 1978; Moreira et al. 2000). In addition, histone H2AX, when phosphorylated on a serine four residues from the carboxyl terminus (serine c‐4), forms γH2AX, which acts as a sensitive molecular marker for DNA damage, specifically double‐strand breaks (DSBs) (Bonner et al. 2008; Ying et al. 2018). DHPN exposure induces DSBs in rat pulmonary epithelial cells, as detected by γH2AX immunoreactivity, indicating it as a suitable DNA damage marker at early stages of rat lung carcinogenesis (Ying et al. 2018). Thus, this study evaluated early γH2AX immunostaining and late development of DHPN‐induced lung and kidney proliferative lesions in female offspring rats whose dams received a low‐protein diet (LPD) during pregnancy.

## 2 Materials and Methods

### 2.1 Experimental Design and Animal Management

Pregnant Wistar rats (F0) were maintained in individual cages in a room under controlled temperature (23°C ± 2°C) and regulated humidity conditions, with periods of 12 h light/dark cycles. Dams received isocaloric chow of a normal protein diet (NPD, 17% protein, *N* = 20) or LPD (6% protein, *N* = 20) during Gestational Day (GD) 1 until parturition (Rizzi et al. 2017). After parturition, all dams were fed NPD until weaning. After the lactation period (21 days of age), female offspring (F1) were randomly distributed among groups (1 female litter/per group): [NPD (*N* = 05), LPD (*N* = 05), NPD + DHPN (*N* = 20), and LPD + DHPN (*N* = 20)], and they were fed NPD for 26 weeks. At 6 weeks old, two groups received 0.2% DHPN (Nakalai Tesque Inc., Kyoto, Japan) in drinking water for 4 weeks (Moreira et al. 2000). Some females (*N* = 05, DHPN groups) were euthanized 24 h after DHPN stopping (first euthanasia), while others (*N* = 15/05, all groups) received only drinking water for an additional 16‐week period (second euthanasia). Food and drinking water were offered ad libitum, and euthanasia was performed by exsanguination under ketamine/xylazine anesthesia. This study received institutional approval from the Ethics Committee for Animal Use of the Bioscience Institute/UNESP (CEUA protocol number #408). In this study, only female offspring were used to provide insights into this underrepresented cohort.

### 2.2 Histological and Immunohistochemistry Analysis

The lung (first and second euthanasia) and kidneys (second euthanasia) were removed, fixed for 24 h in 10% buffered formalin, histologically processed, and paraffin‐embedded. Five‐micrometer lung tissue sections were used for γH2AX immunohistochemical staining (DHPN groups, *N* = 05), or lung and kidney sections were used for conventional hematoxylin and eosin (HE) staining (all groups). Rat lung and kidney preneoplastic and neoplastic lesions were classified according to previously published criteria (Renne et al. 2009; Frazier et al. 2012). Lung tissue sections (first euthanasia) were placed on silanized‐coated slides, deparaffinized, and rehydrated with graded alcohol. These sections were subjected to Pascal pressure chamber antigen retrieval in a citrate acid buffer at pH 6.0 for 30 min at 120°C. Endogenous peroxidase was blocked with 3% H_2_O_2_ in PBS for 10 min in the dark. After washing with phosphate‐buffered saline (PBS), slides were incubated with primary antibody anti‐γH2AX, 1:200 dilution (Invitrogen, USA) in a humidified chamber (overnight, at 4°C) (Zapaterini et al. 2022). Then, the slides were incubated with one‐step horseradish peroxidase polymer (EasyPath–Erviegas, Brazil) (20 min). The immunoreaction was visualized with 3‐diaminobenzidine chromogen (Sigma–Aldrich, USA) and counterstained with Harris hematoxylin. γH2AX labeling indices (positive cells per lung area) were calculated from measurements of at least five randomly acquired non‐overlapping fields, 1 section/animal (×20 objective), using Image J software (NIH, USA). Epithelial cells lining the bronchioles and alveoli were evaluated, while alveolar macrophages and other nonalveolar cells were excluded from the γH2Ax analysis.

### 2.3 Statistical Analysis

Data from body weight, lung and kidney weights, liquid consumption (DHPN exposure period), γH2AX immunoreactivity, and the mean number of proliferative lesions were analyzed by Student's *t*‐test or Mann–Whitney test (two‐group comparison) or ANOVA or Kruskal–Wallis test (four‐group comparison). The percentage of rats bearing proliferative lesions (%) was analyzed using the *χ* ^2^ test. Statistical analysis was performed in GraphPad Prism software (version 8.01; La Jolla, CA, USA). Significant differences were assumed when *p* < 0.05.

## 3 Results and Discussion

There was no significant difference in mean values of maternal body weight, food consumption, litter size, or gender distribution between LPD and NPD groups (data not shown). At birth, mean body weight from litters of female offspring whose dams were fed LPD was significantly lower than litters of female offspring whose dams were fed NPD (5.03 ± 0.60 vs. 6.24 ± 0.47 g, *p* < 0.0001). At weaning, mean values of body weight of female litters from the LPD group remained significantly lower (44.23 ± 4.76 vs. 52.60 ± 5.93 g, *p* < 0.0001) than those of litters from the NPD group. These findings of low body weight at parturition and weaning of pups from dams receiving LPD have been previously described (Rizzi et al. 2017; Sene et al. 2018; Rinaldi et al. 2013; Pinho et al. 2014; Assalin et al. 2019; Folguieri et al. 2022).

During Weeks 6–10, the two groups that received 0.2% DHPN in drinking water showed similar liquid intake but significantly diminished (*p* < 0.0001) when compared with their control groups receiving only drinking water (Table 1). At the end of Week 10, the mean body weights of female offspring groups after 0.2% DHPN exposure were significantly lower (*p* < 0.0001) than those of female offspring receiving only drinking water (Table 1). These findings of lower mean values for liquid intake and body weight induced by this carcinogen regimen have been previously observed (Moreira et al. 2000), which might be related to mild systemic DMH toxicity and unpalatability (Moreira et al. 2000; Okamura et al. 2004). The γH2AX labeling indices in the lung tissue from the LPD + DHPN group were increased significantly (*p* < 0.0021) in relation to the NPD + DHPN group at 24 h after DHPN stopping (Figure 1A,B and Table 1). Ying et al. (2018) observed a significant increase in γH2AX‐positive pulmonary cells at 0, 1, 3, and 7 days after 0.1% DHPN exposure (2 weeks) in male F344 rats. In our previous study (Zapaterini et al. 2022), we showed that maternal LPD, during gestation and lactation periods, deregulated 21 genes related to DNA repair and DNA replication pathways in the mammary gland of the LPD female offspring (F1) following a single dose of MNU carcinogen. These findings were confirmed by an increase in γH2AX‐positive epithelial cells in the mammary gland tissue from the LPD group, 24 h after MNU administration. Furthermore, these early postnatal events were followed by significantly higher susceptibility to mammary carcinogenesis in offspring during adulthood. Thus, higher DNA damage level in pulmonary epithelial cells, probably associated with lower DNA repair capacity during DHPN exposure, could also have contributed to increased lung carcinogenesis in female offspring whose dams received a gestational LPD.

**TABLE 1:** Effects of gestational low protein diet on body weight, γH2AX labeling indices, lung and kidney weights, and histological findings in DHPN‐induced carcinogenesis in female Wistar rats at Week 26.

Parameters/groups	NPD + DHPN	LPD + DHPN	NPD	LPD
Weeks 3–10				
Number of female rats	20	20	05	05
Liquid intake (mL/rat/day)^a^	17.40 ± 1.42^e^	18.02 ± 1.60^e^	20.64 ± 1.05	21.70 ± 1.22
Body weight at Week 10 (g)	185.27 ± 14.23^e^	181.07 ± 12.54^e^	205.17 ± 15.12	207.33 ± 13.94
γH2AX LI (10 × 10^−3^ mm^2^)	9.19 ± 2.06	16.52 ± 3.65^e^	—	—
Week 26				
Number of female rats	15	15	05	05
Body weight at Week 26 (g)	360.27 ± 22.52	364.67 ± 24.57	358.17 ± 24.38	368.17 ± 33.59
Lung weight (g)	2.09 ± 0.15	1.95 ± 0.18	2.00 ± 0.11	1.99 ± 0.12
Lung weight (%)	0.58 ± 0.06	0.54 ± 0.07	0.57 ± 0.07	0.55 ± 0.04
Kidney weight (g)	1.68 ± 0.14	1.71 ± 0.13	1.66 ± 0.09	1.69 ± 0.22
Kidney weight (%)	0.47 ± 0.05	0.47 ± 0.06	0.46 ± 0.02	0.46 ± 0.08
*Histological findings*				
Lung BAH				
Incidence (%)^b^	100% (15)^d^	100% (15)	0	0
Mean number/rat	3.07 ± 1.39 (46)	4.67 ± 2.16 (70)^e^	0	0
Lung adenomas				
Incidence (%)^b^	73% (11)	100% (15)	0	0
Mean number/rat	0.87 ± 0.64 (13)^c^	2.00 ± 1.25 (30)^e^	0	0
Mean size (mm^2^)^c^	0.12 [0.06–0.23]	0.14 [0.05–0.31]	0	0
Kidney ATH				
Incidence (%)^b^	80% (12)	87% (13)	0	0
Mean number/rat^c^	1.0 [1.0–2.0] (21)	2.0 [1.0–3.0] (29)	0	0

Abbreviations: ATH = atypical tubule hyperplasia; BAH = bronchioloalveolar hyperplasia; DHPN = 0.2% *N*‐bis(2‐hydroxypropyl)nitrosamine in drinking water for 4 weeks was offered to female offspring during Weeks 6–10; LI = labeling indices per lung area (*n* = 05 each group) and LPD = low protein diet containing 6% protein were offered to dams during gestation; NPD = normal protein diet containing 17% protein.

^a^ Liquid intake during Weeks 6–10.

^b^ Incidences are expressed as percentage of animals bearing lesions.

^c^ Median and 25% and 75% percentile.

^d^ Total number animals or histological lesions detected.

^e^ Different from respective control groups, 0.022 < *p* < 0.0001.

**FIGURE 1:**
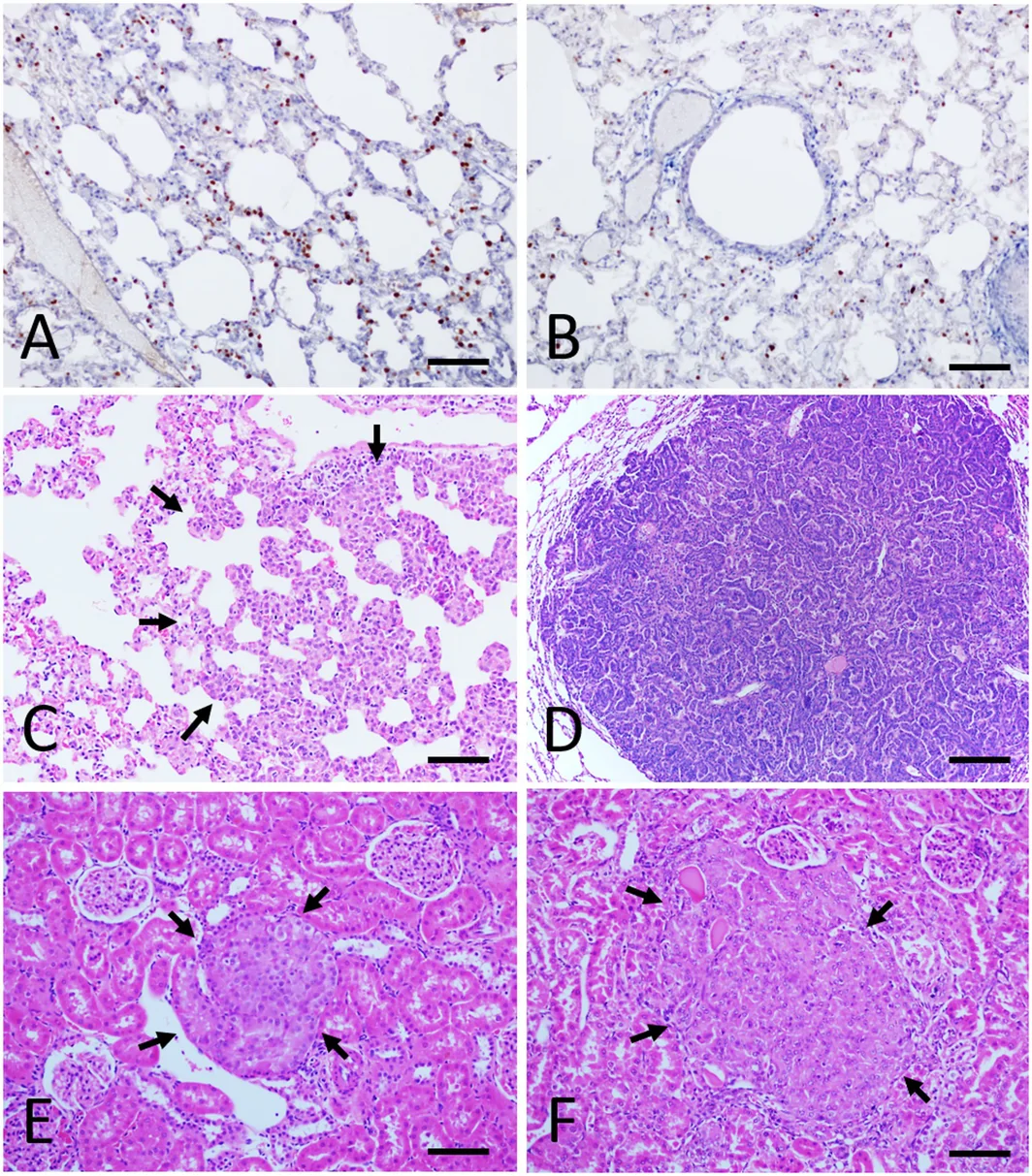
(A, B) Representative lung immunostaining for anti‐γH2AX antibody (brown nuclei). (C, D) Representative HE‐stained lung sections from bronchioloalveolar hyperplasia (BAH) (arrows) and adenoma induced by *N*‐bis(2‐hydroxypropyl)nitrosamine (DHPN). (E, F) Representative HE‐stained kidney sections from atypical tubule hyperplasia (ATH) (arrows) induced by *N*‐bis(2‐hydroxypropyl)nitrosamine (DHPN). Bar = 50 μm (A, B, C, E, and F) or 100 μm (D). A, D, and E—LPN + DHPN group; B, C, and F—NPD + DHPN group.

The findings on body weight, organ weights, and lung and kidney proliferative lesions at week 26 are shown in Table 1. The mean values of final body weight and lung and kidney weights (absolute and relative) indicated no differences among groups. In the DHPN‐exposed groups, lung lesions such as bronchioloalveolar hyperplasia (BAH) and adenoma were observed (Figure 1C,D and Table 1). The mean number of BAH/rat and the number of bronchioloalveolar adenomas/rat were significantly increased (*p <* 0.022 and *p* < 0.007, respectively) in the lung of LPD + DHPN group in comparison to their counterpart group (Table 1). However, there was no statistically significant difference in the incidence of pulmonary proliferative lesions or the mean size of adenomas between DHPN groups (Table 1). In addition, neither tumor was diagnosed, nor a statistical difference in incidence or mean number of atypical tubule hyperplasia (ATH) were observed in the kidneys between DHPN groups (Table 1). Furthermore, no lung or kidney proliferative lesion was observed in the NPD or LPD groups. These results suggest that, from the perspective of lung carcinogenesis, in utero LPD increases the occurrence of DHPN‐induced proliferative lesions.

Using female Wistar rats, a 26‐week medium‐term DHPN carcinogenesis model proved insufficient to induce malignant lung (carcinomas) or kidney tumors (adenomas and carcinomas) following a 4‐week exposure to 0.2% DHPN. In fact, the lung tumors identified in this study were classified as bronchioloalveolar adenomas based on the absence of cytological and histological features of malignancy, including cellular and nuclear atypia, increased mitotic activity or atypical mitosis, invasion of adjacent structures, and destruction of the airway walls (Renne et al. 2009) (Supplementary Data). Establishing a clear dose–response in DHPN carcinogenesis typically requires a long‐term protocol (Konishi et al. 1978; Moreira et al. 2000). It also depends on rat strain susceptibility and sex‐related effects, given that DHPN actively induces benign and malignant tumors across a wide spectrum of organs. In general, F344 rats exhibit greater susceptibility to DHPN‐induced carcinogenesis than SD and Wistar rats, with males showing higher vulnerability than females, particularly in target organs such as the lung, thyroid, and kidney (Konishi et al. 1978; Moreira et al. 2000). Thus, a long‐term evaluation is required to effectively assess the effects of maternal LPD on DHPN‐induced carcinogenesis.

In conclusion, gestational low protein (GLP) alone does not directly induce lung tumors but significantly enhances the development of DHPN‐induced lung proliferative lesions in female offspring at Week 26. Furthermore, this study reveals that the increase in γH2AX positivity observed after 24 h of carcinogen exposure, used here as a biomarker for genotoxicity analysis (Plappert‐Helbig et al. 2019), may be a suitable molecular mechanism by which GLP increases the risk of development of lung proliferative lesions in adult female offspring exposed to DHPN.

## Author Contributions

V.d.C.M.P.F. contributed to methodology, writing and editing. V.H.C. contributed to data curation, writing and editing. P.A.B. contributed to methodology, writing and editing. L.F.B. contributed to conceptualization, project administration, funding acquisition, writing and editing. All authors contributed to the article and approved the submitted version.

## Funding

This study was supported by the National Council for Scientific and Technological Development (CNPq) (Grants 400563/2021‐5; V.d.C.M.P.F. and 304093/2024‐6; L.F.B.).

## Conflicts of Interest

The authors declare no conflicts of interest.

## Supporting information


**Data S1:** em70086‐sup‐0001‐Supinfo1.pdf.

## Data Availability

The data that support the findings of this study are available from the corresponding author upon reasonable request.
