# Supplementary material for: Gestational Low Protein Diet Increases the Susceptibility to N‐Bis(2‐Hydroxypropyl)nitrosamine‐Induced Lung Tumors in Female Wistar Rats
Source: Environ Mol Mutagen. 2026 Oct 24;67(8-9):e70086. doi: 10.1002/em.70086 (PMC13695019; doi:10.1002/em.70086)
Supplement: Supplementary file 1 [file EM-67-0-s001.pdf]

Gestational low protein diet increases the susceptibility to N-bis(2-hydroxypropyl)nitrosamine-induced lung tumors in female Wistar Rats.

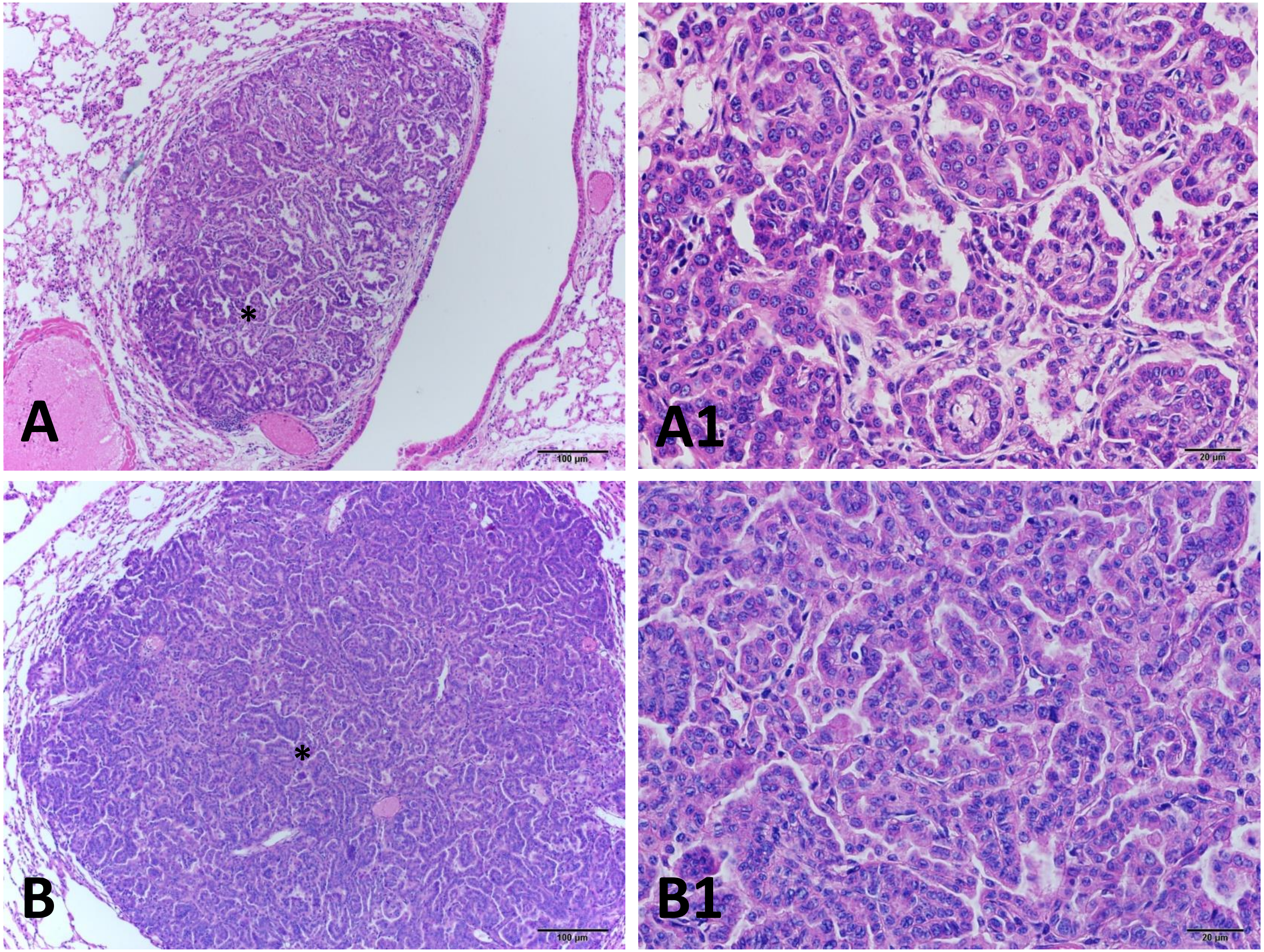

A and B = Representative HE-stained lung sections from bronchioloalveolar adenoma induced by N-bis(2-hydroxypropyl)nitrosamine (DHPN) in normal-protein and low-protein diet groups, respectively. The areas marked with asterisk in the left panels (A and B) are shown at higher magnification in the corresponding right panels (A1 and B1).
